# Tumorigenic potential of pituitary tumor transforming gene (PTTG) *in vivo* investigated using a transgenic mouse model, and effects of cross breeding with p53 (+/−) transgenic mice

**DOI:** 10.1186/1471-2407-12-532

**Published:** 2012-11-20

**Authors:** Miranda Y Fong, Hanan Farghaly, Sham S Kakar

**Affiliations:** 1 Department of Physiology and Biophysics, University of Louisville, 505 South Hancock Street, CTRB 322, Louisville, KY, 40202, USA; 2 Department of Pathology, University of Louisville, Louisville, KY, 40202, USA; 3 Molecular Targets Program, James Graham Brown Cancer Center, University of Louisville, Louisville, KY, 40202, USA

**Keywords:** PTTG, Transgenic mice, p53, Tumorigenesis, Cancer

## Abstract

**Background:**

Pituitary tumor-transforming gene (PTTG) is an oncogene that is overexpressed in variety of tumors and exhibits characteristics of a transforming gene. Previous transgenic mouse models to access the tumorigenic potential in the pituitary and ovary have resulted in dysplasia without formation of visible tumors, possibly due to the insufficient expression of PTTG. PTTG expression level is critical for ovarian tumorigenesis in a xenograft model. Therefore, the tumorigenic function of PTTG *in vivo* remains unclear. We generated a transgenic mouse that overexpresses PTTG driven by the CMV promoter to determine whether PTTG functions as a transforming oncogene that is capable of initiating tumorigenesis.

**Methods:**

Transgenic animals were generated by microinjection of PTTG transgene into the male pronucleus of FVB 0.5 day old embryos. Expression levels of PTTG in tissues of transgenic animals were analyzed using an immunohistochemical analysis. H&E staining and immunohistostaining were performed to examine the type of tumor in transgenic and PTTG transgenic/p53^+/-^ animals.

**Results:**

PTTG transgenic offspring (TgPTTG) were monitored for tumor development at various ages. H&E analysis was performed to identify the presence of cancer and hyperplastic conditions verified with the proliferation marker PCNA and the microvessel marker CD31. Immunohistochemistry was performed to determine transgene expression, revealing localization to the epithelium of the fallopian tube, with more generalized expression in the liver, lung, kidney, and spleen. At eight months of age, 2 out of 15 TgPTTG developed ovarian cancer, 2 out of 15 developed benign tumors, 2 out of 15 developed cervical dysplasia, and 3 out of 15 developed adenomyosis of the uterus. At ten months of age, 2 out of 10 TgPTTG developed adenocarcinoma of the ovary, 1 out of 10 developed a papillary serous adenocarcinoma, and 2 out of 10 presented with atypia of ovarian epithelial cells. Tumorigenesis is a multi-step process, often requiring multiple oncogenes and/or inactivation of tumor suppressor genes. Therefore, to understand the contribution of p53 to PTTG induced tumorigenesis, we crossbred TgPTTG to p53^+/−^ mice and maintained those 8 to 10 months. TgPTTG/p53^+/−^ animals developed sarcomas faster than p53^+/−^ alone as well as different tumor types in addition to cervical carcinomas *in situ* in 10 out of 17 females.

**Conclusions:**

We conclude that while PTTG is a functional transforming oncogene, it requires an additional partner to effectively promote tumorigenesis through the loss of p53 include or between function or modulation.

## Background

The pituitary tumor transforming gene (PTTG), also known as securin, is ubiquitously expressed at a low basal level where it functions in regulating sister chromatid separation [1]. Its physiologic functions include cell proliferation, ensuring the fidelity of DNA replication, DNA damage repair, organ development, and metabolism [2]. Overexpression of PTTG influences multiple pathways for cancer initiation and progression including enhanced cell proliferation [3], genomic instability [4], and cellular transformation [3,5]. Its transforming ability has been demonstrated *in vitro* where over-expression of PTTG induces anchorage-independent growth in soft agar and *in vivo* xenograft tumor formation in nude mice using rat fibroblast NIH3T3 cells and human embryonic kidney HEK293 cells [3,5]. PTTG overexpression has been correlated with the promotion of angiogenesis through increased expression and secretion of several factors including basic fibroblast growth factor (bFGF), vascular endothelial growth factor (VEGF), and interleukin 8 (IL-8) [3,6,7]. PTTG is implicated in metastasis through the induction of the epithelial to mesenchymal transition [8,9].

PTTG overexpression has been identified in a variety of endocrine-related tumors, including pituitary, ovarian, uterine, breast, and thyroid [5,6,10,11] and non-endocrine related tumors such as lung, gastrointestinal, and gliomas. PTTG expression is also detected in germ cell tumors, sex-cord and stromal cell tumors, epithelial tumors arising from the ovary and in multiple types of breast cancer, including invasive ductal carcinomas, ductal *in situ* carcinomas, and infiltrating ductal carcinomas [11]. In pituitary adenomas, PTTG is implicated in tumor initiation and progression [12]. It has also been identified as an oncogene in pituitary tumors activated in the early stages of cellular transformation, from normal to hyperplastic [13], and has been correlated with tumor invasiveness [6]. Levels of PTTG expression have also been correlated to the degree of malignancy, pathogenesis, and progression of colorectal, thyroid, and breast tumors [14-16]. In the case of gliomas, PTTG has been correlated to poor prognosis in patients [17]. PTTG is abundantly expressed in several carcinoma cell lines including cervical carcinoma HeLa cells, choriocarcinomas JEG-3 and JAR, breast adenocarcinoma MCF-7, osteogenic sarcoma U-2OS, hepatocellular carcinoma Hep 3B, lung carcinoma H1299, EY and A549, ovarian CAOV3 and A2780, and thyroid carcinoma TC-1 [18,19]. These finding indicate that PTTG may be involved in transformation of several tissues leading to tumorigenesis.

Transgenic PTTG^−/−^ mice exhibit pituitary hypoplasia and, upon cross-breeding with heterozygous deletion of retinoblastoma (Rb^+/−^), show a tumor development rate of 30%. Comparatively Rb^+/−^/PTTG^+/+^ develop tumors at 86% by 13 months of age [20]. PTTG silencing using siRNA on xenograft tumors from an ovarian cancer cell line and hepatocellular carcinoma cell line reduced both the size and incidence of tumor burden; however, incomplete silencing of PTTG led to a reduction of tumor burden, while complete silencing showed nearly complete eradication of tumors, indicating that PTTG expression impacts tumor formation and tumor growth [19,21].

Previously, our lab developed a transgenic mouse model that over-expresses human PTTG cDNA under the control of Müllerian inhibiting substance type II receptor (MISIIR). These mice presented with an increased mass of the corpus luteum as well as an increase in serum LH and testosterone, but failed to generate visible ovarian tumors [22], possibly due to a weak promoter that was unable to produce the required level of PTTG protein to initiate tumorigenesis. In addition, Abbud *et al*. [23] generated a PTTG transgenic mouse under the control of the alpha-subunit of glycoprotein hormone (αGSU) to target expression to the gonadotroph cells of the pituitary, resulting in gonadroph hyperplasia and microadenomas with plurihormal hyperplasia, accompanied by prostatic and seminal vesicle hyperplasia. Tumorigenesis often requires multiple gene mutations including activation or amplification of oncogenes, increased growth factors and their receptors, and/or inactivation of tumor suppressor genes. As such, PTTG expression by itself may not be sufficient to drive tumorigenesis and may require a partner gene [24]. Therefore, in our current study, we have selected the CMV promoter to drive expression of human PTTG cDNA to produce PTTG transgenic (TgPTTG) mice to understand the tumorigenic potential of PTTG *in vivo*. In addition, we crossbred TgPTTG with p53^+/−^ mice. TgPTTG mice developed ovarian adenocarcinomas and adenocarcinoma of the fallopian tube. Crossbreeding of TgPTTG with p53^+/−^ mice resulted in enhanced tumor incidence, earlier tumor formation, and carcinomas *in situ* of the cervix.

## Methods

### Construction of CMV-PTTG-EGFP transgene

**Figure 1:**
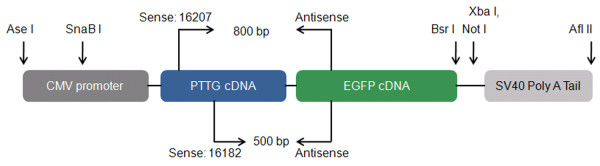
**Construct of PTTG**-**EGFP transgene with CMV promoter with primer annealing sites and restriction enzyme sites.**

Human PTTG cDNA was subcloned into the multiple cloning site of the pEGFP-N3 vector containing the CMV promoter and EGFP reporter gene. The vector was restricted using Ase I and Afl II restriction enzymes (New England BioLabs) by incubating with the vector for 2 h at 37°C. The restricted vector was run on a 0.7% agarose gel to isolate the transgene fragment, CMV-PTTG-EGFP (Figure 1). CMV-PTTG-EGFP was excised from the gel and purified using a gel extraction kit (Qiagen). CMV-PTTG-EGFP was then sequenced to test the authenticity of the sequence.

### Animal housing

Mice were housed in a conventional facility with a 12 h light: 12 h darkness cycle and fed standard chow and water ad libdum. All animals were treated in accordance with National Institutes of Health Guidelines for the Care and Use of Laboratory Animals and approved by Institutional Animal Care and Use Committee (IACUC) at the University of Louisville.

### Generation of transgenic animals

Transgenic animals were generated in association with the University of Cincinnati Transgenic Mouse Core Facility. Transgene DNA was microinjected into the male pronucleus of FVB 0.5 day old embryos. Embryos were then transplanted into a pseudo-pregnant female. Wild type males were bred to positive TgPTTG female founders. TgPTTG F_1_ males were bred to wild type females to establish a colony line.

### P53^+/−^ Mice

P53^+/−^ mice on an FVB background were obtained from Jackson Laboratory. Female p53^+/−^ were crossbred with male TgPTTG mice from the same founder line (#71309) to generate TgPTTG/p53^+/−^ mice.

### Genotyping and screening of transgenic and p53^+/−^ mice

Mice were tail clipped between 21–28 days of age and toe tattooed with an ID number. DNA from tail clips was extracted using PCR Extract-N-Amp kit (Sigma). PTTG-EGFP genotype was identified via PCR using the specific primer: PTTG 16182: sense 5’-ACT GAG AAG ACT GTT AAA GC-3’ or PTTG 16207: sense 5’-ACG AAT TCA TGG CTA CTC TGA TCT ATG T-3’, and EGFP antisense 23759: 5’- AGA TGA ACT TCA GGG TCA GC-3’ that specifically amplified the transgene sequence (Figure 1). Two PTTG sense primers were used to verify accuracy of amplification. PCR conditions were 1) 94°C for 5 min, 2) 94°C for 30 sec, 3) 58°C for 30 sec, 4) 72°C for 1 min, 5) steps 2–4 were repeated for 30 cycles, 6) 72°C for 7 min. P53^+/−^ genotype was identified via PCR using the specific primers according to Jackson Laboratory protocol: Wild type: sense 5’-ACA GCG TGG TAC CTT AT-3’, Common: antisense 5’- TAT ACT CAG AGC CGG CCT -3’, and Mutant: sense 5’-CTA TCA GGA CAT AGC GTT GG-3’. PCR conditions were 1) 94°C for 3 min, 2) 94°C for 30 sec, 3) 64°C for 1 min, 4) 72°C for 1.5 min, 5) Steps 2–4 repeated for 35 cycles, 6) 72°C for 2 min.

### RNA isolation and analysis of transgene expression via RT-PCR

Tissues were preserved in RNA Later (Sigma) at the time of sacrificing. Tissues were homogenized in 1 ml of Trizol (Sigma) and isolated via standard procedures. Total RNA was quantitated by NanoDrop. One μg of total RNA was converted to cDNA using a reverse transcription kit (BioRad). Total cDNA was then subjected to PCR amplification as described above.

### Histological analysis

Tissues samples were fixed in 10% neutral buffered formalin (Fisher Scientific) overnight at RT. After 24 h, formalin was then replaced with 70% ethanol and the tissues were stored at 4°C until processing. Tissues were embedded in paraffin using standard techniques. Five micrometer sections were stained with H&E by the Pathology Core Research Laboratory, University of Louisville and evaluated by a trained pathologist, Hanan Farghaly, MD.

### Immunofluorescence

Formalin-fixed paraffin embedded tissue sections were deparaffinized in fresh xylene and rehydrated in a graded series of ethanol. Antigen retrieval was conducted by incubating the slides in 10 mM sodium citrate (pH 6.0) at 95°C for 20 min then rinsing twice with PBS. Slides were incubated in 4 drops per section of Image-It FX Signal Enhancer (Invitrogen) for 30 min in a humidity chamber and then rinsed with PBS. Slides were blocked with 10% goat serum (Sigma) in PBS for 1 h followed by anti-PTTG diluted 1:1,500 in PBS and incubated at 4°C overnight. Slides were then washed in PBS before application of a secondary Alexa 594 labeled anti-rabbit (Invitrogen) diluted 1:500 in PBS containing 1 drop of goat serum per 5 ml and incubated for 45 min at RT in the dark, then washed three times with PBS. Images were acquired on Nikon Eclipse E400 and ACT-1.1 imaging software (Huntley, IL, USA).

### Immunohistochemistry

Formalin-fixed paraffin embedded tissues were deparaffinized in xylene and rehydrated in a decreasing graded series of ethanol. Antigen retrieval was conducted by incubating the slides in 10 mM sodium citrate (pH 6.0) at 95°C for 20 min, then rinsed in PBS for 5 min. Slides were incubated with 0.3% hydrogen peroxide in methanol for 20 min at RT and rinsed three times in PBS for 5 min each. For proliferating cell nuclear antigen (PCNA) expression, slides were blocked using a Mouse-On-Mouse (M.O.M.) peroxidase kit (Vector Laboratories) for 1 h at RT. Blocking solution was poured off and slides were incubated in M.O.M. diluent for 5 minutes. PCNA diluted 1:2,000 (Cell Signaling) in SignalStain antibody diluent (Cell Signaling) was incubated overnight at 4°C. After washing three times in PBS for 5 min each, secondary anti-mouse biotinylated IgG from M.O.M. kit was incubated for 45 min at RT followed by 30 min incubation with streptavidin. After three washes in PBS for 5 min each, 3, 3’-diaminobenzidine (DAB, Vector Laboratories) was used to develop color. For CD31, slides were blocked using Vectastain anti-rabbit Elite ABC kit (Vector Laboratories) for 1 h at RT and then incubated with anti-CD31 (1:50, AbCam) in PBS overnight at 4°C. Vectastain anti-rabbit Elite ABC kit (Vector Laboratories) and DAB was used to develop color.

## Results

### Generation of CMV-PTTG-EGFP transgenic mice

**Figure 2:**
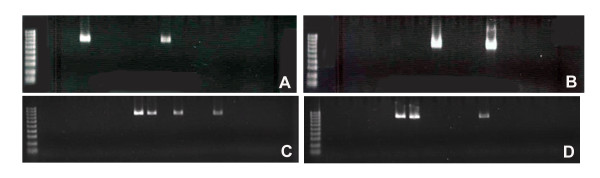
**Genotyping of transgenic mice by PCR using sense PTTG primer 16207 and EGFP antisense primer**. (**A**-**B**) founders, (**C**-**D**) a representative genotyping of offspring. Lane 1, 1 kb ladder; lane 2, blank; lanes 3-end, individual mice.

In previous attempts to generate TgPTTG under the control of promoters MISIIR and αGSU, mice did not develop visible ovarian or pituitary tumors [22,23]. Therefore, we asked whether the level of PTTG expression was sufficient to drive tumorigenesis as the level of PTTG expression was shown to be significant for tumor growth [19,21]. To this end we selected the non-specific strong CMV promoter to drive PTTG expression. Human PTTG cDNA was subcloned into the multiple cloning site of the N_3_ expression vector containing the CMV promoter and a downstream enhanced GFP (EGFP) marker gene. In order to reduce the transgene fragment size for ideal transgenic mouse production, the N_3_-PTTG was restricted with Ase I and Afl II restriction enzymes, resulting in the transgene fragment CMV-PTTG-EGFP (Figure 1). The transgene was sequenced to ensure authenticity of the sequence. EGFP has been shown not to impact target gene function [25]. The transgene was microinjected into the male pronuclei of 0.5 day old Friend Virus B-Type (FVB) embryos. TgPTTG founders were identified by PCR screening of DNA extracted from the tail clip of 34 F_0_ mice using two different sense primers located in PTTG and one antisense primer located in EGFP (Figure 1). This resulted in the identification of 1 male (#71288) and 3 female TgPTTG founders (#71282, 71305, and 71309; Figure 2A-B). The founders were crossbred with wild type (WT) FVB mice and resultant F_1_ offspring were genotyped by PCR (Figure 2C-D). All founders were fertile and produced an average litter size (Table 1). However, the male and 1 female founder (#71282) failed to transmit the transgene to their offspring (F_1_), suggesting that the transgene did not integrate into the germ line. F_1_ TgPTTG males from a single line (founder #71309) were crossbred with WT females through multiple generations to create F_2_-F_4_ to generate a sufficient number of TgPTTG mice. TgPTTG males were often aggressive by 6 months of age and had to be maintained in separate cages. All mice demonstrated hyperactivity during housing.

**Table 1:** Breeding summary of TgPTTG founders

**Founder**	**Number of breeding**	**Total number of offspring**	**Number of positive mice in F1**
71282	5 (WT)	37	None
71288	5 (WT)	43	None
71305	5 (WT)	42	16
71309	4 (WT)	36	19

WT = wild type.

### PTTG expression in CMV-PTTG-EGFP mice

**Figure 3:**
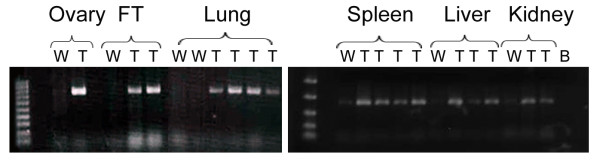
**Reverse**-**transcription PCR of specified tissues from WT** (**W**) **and transgenic** (**T**) **mice.** Lane 1, ladder; lane 2, empty; lanes 3-end, samples. B = blank negative control of reaction mix without template. FT = fallopian tube.

TgPTTG mice were sacrificed at 4, 6, 8, and 10 months of age along with the age-matched WT controls and tissues were harvested for RNA isolation and immunohistochemistry. Tissues selections were determined by promoter expression [26] and examined by reverse transcription/PCR of RNA isolated from the ovary, fallopian tube, lung, spleen, liver, and kidney (Figure 3). As expected, PTTG expression was detected in all of the tissues.

**Figure 4:**
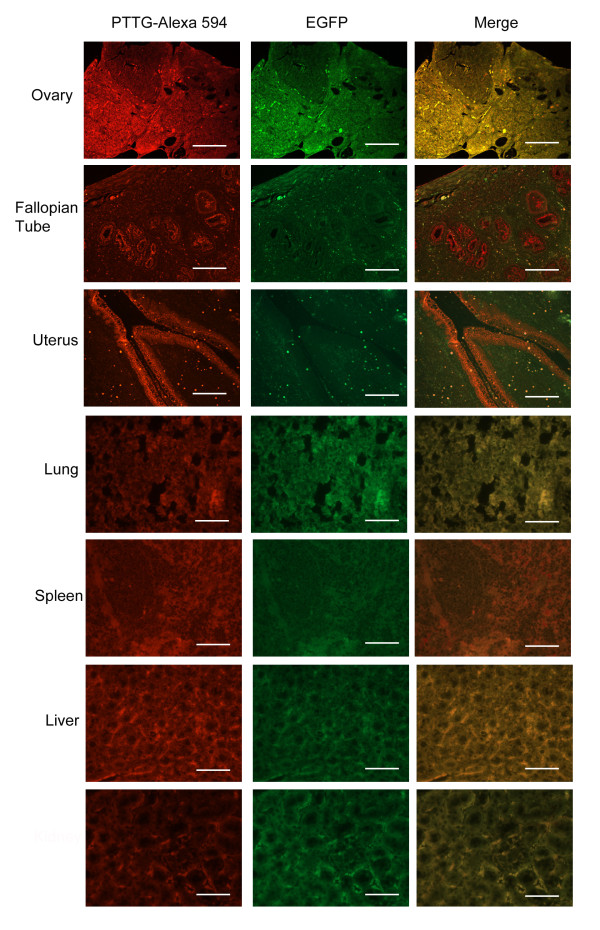
**Immunofluoroscence of TgPTTG mice for PTTG**-**EGFP protein expression.** PTTG antibody was used for immunostaining of PTTG protein and detected with Alexa 594 fluorescent antibody (red). The same field viewed with a FITC filter (GFP) and the images were merged. White bar indicates 50 μm.

Protein expression of the transgene was determined by immunofluorescence to determine if expression was localized to any specific cell type or more broadly distributed. PTTG primary antibody raised in our laboratory [27] was detected with Alexa 594 secondary antibody (red). Sections were then analyzed by fluorescence microscopy using a Texas Red filter for PTTG expression and GFP filter for EGFP expression. Specificity of the PTTG antibody to human PTTG vs. murine PTTG has been addressed previously [22]. Immunofluorescence of the fallopian tube and uterus showed intense staining in the epithelium, while in the ovary, staining was observed in stromal cells (Figure 4) in 4 months old animals. Lung, spleen, liver, and kidney showed general non-specific expression of the transgene.

### Histology and immunohistochemistry of TgPTTG tissues

**Figure 5:**
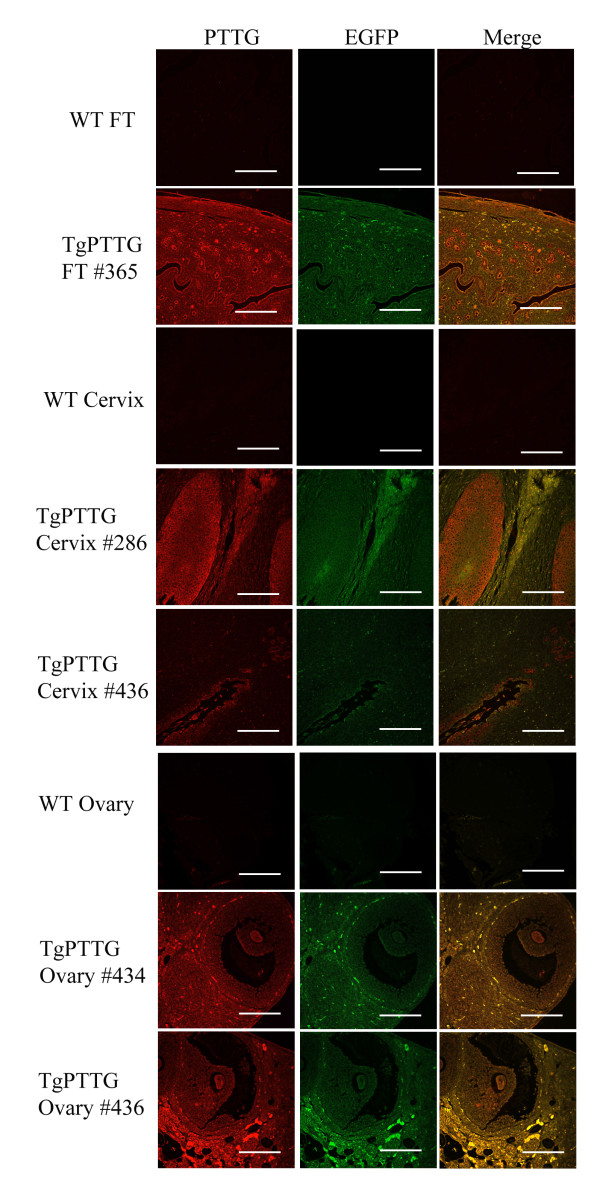
**Immunofluorescence of TgPTTG 8 months old mice for PTTG**-**EGFP protein expression.** PTTG antibody was used for immunostaining of PTTG protein and detected with Alexa 594 fluorescent antibody (red). Images were acquired using confocal microscopy. WT = wild type, FT = fallopian tube. White bar indicates 50 μm.

**Figure 6:**
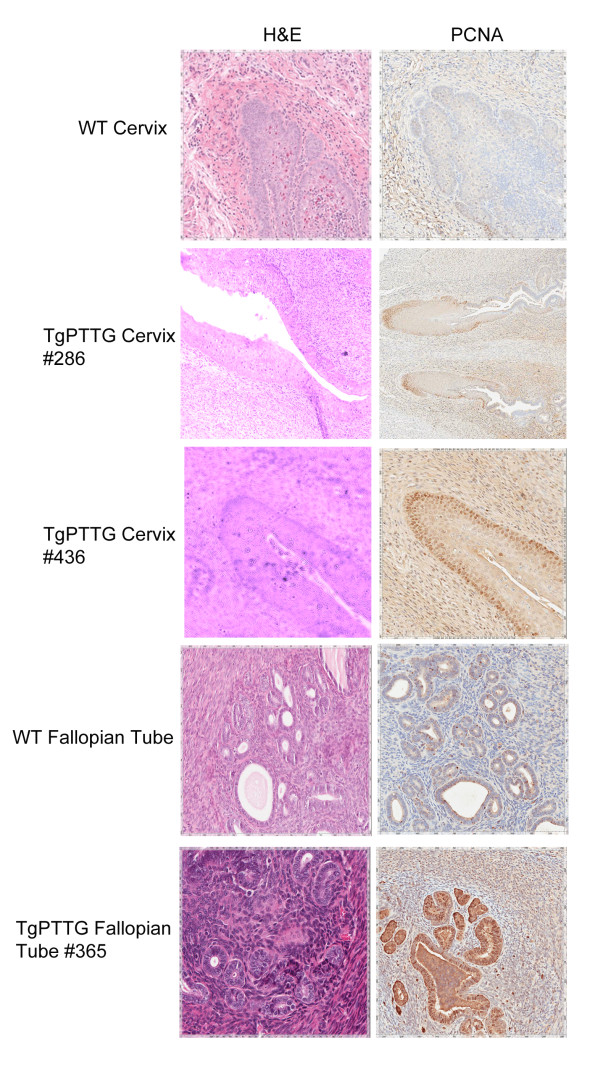
**Immunohistochemistry of 8 months TgPTTG mice with pre**-**cancer conditions compared to age**-**matched WT mice.** PCNA antibody was incubated overnight and then visualized with DAB (brown color).

**Figure 7:**
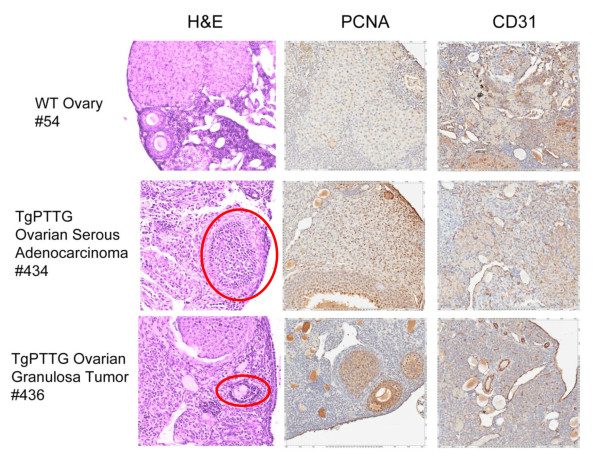
**Immunohistochemistry of 8 months TgPTTG mice with cancer compared to age**-**matched WT mice.** PCNA and CD31 antibodies were incubated overnight and then visualized with DAB (brown color). Red circle indicates *in situ* tumor.

Formalin-fixed paraffin embedded tissues were sectioned and stained with hematoxylin and counterstained with eosin (H&E). Evaluation of the histology was performed by a licensed pathologist (HF). Tissues were selected based on CMV promoter expression [26] to include liver, kidney, lung, and spleen as well as additional tissues of interest including ovary, fallopian tube, and uterus. While all tissues were normal in 4 and 6 months old TgPTTG mice, pre-cancerous conditions (3 out of 15, 20%) and carcinomas (2 out of 15, 13.3%) were observed at 8 months of age. A total of 15 females were included in the TgPTTG 8 month old group and were compared with 11 WT age-matched controls. PTTG-EGFP expression was confirmed by immunofluorescence (Figure 5). Pronounced follicular cysts and corpus luteum were observed in 6 out of 15 (40%) of TgPTTG females. Pre-cancerous conditions included one female (#286) having squamous dysplasia with extensive keratinization at the transformation zone between the uterus and the cervix (Figure 6), a second female (#365) developed hyperplasia of the fallopian tube with endothelial atypia (Figure 6), while a third female (#436) developed low grade dysplasia of squamous epithelium with acute cervicitis (Figure 6). Inflammation was also observed in additional females, predominately in the cervix (2 out of 15, 13.3%) and fallopian tube causing dilation (3 out of 15, 20%). Hyperplastic and dysplastic conditions were confirmed using PCNA staining compared to age-matched controls [28]. PCNA staining for TgPTTG #286 revealed 5% positive cells with reverse maturation of squamous epithelium of the cervix. TgPTTG #365 was 25% positive for PCNA and localized to the epithelial glands of the fallopian tube. PCNA staining for TgPTTG #436 cervix was 5-10% positive, localizing to the basal cells and showed a pattern of reverse maturation. Age-matched WT fallopian tubes and cervix were negative for PCNA demonstrating that PTTG increased proliferation. Three females (20%) developed adenomyosis of the uterus. An additional two females (13.3%) developed ovarian cancer (#434, 436; Figure 7). PCNA and CD31 staining were performed to compare proliferation and angiogenesis in TgPTTG ovarian carcinomas compared to age-matched WT animals. We decided to use a rating system for CD31 staining, where 1+ means < 25% of tissue was stained, 2+ means 25-75% of tissue was stained, and 3+ means > 75% of tissue was stained in accordance with pathology standards. TgPTTG #434 developed ovarian serous adenocarcinoma with PCNA staining showed 35% positive area and CD31 staining was 2+ (Figure 7). TgPTTG #436 developed a granulosa cell tumor and a follicular cyst with mitotic figures. In this female, PCNA staining was 5% and CD31 staining was 2+ (Figure 7). Comparatively, age-matched WT animals showed 1% PCNA staining of the ovary, which was localized to the granulosa cells of the follicle as expected, while CD31 staining was 1+ (Figure 7). A summary of PCNA and CD31 staining patterns are reported in Table 2. All other tissues were devoid of visible tumors.

**Table 2:** Summary of PCNA and CD31 staining in 8 months old WT and TgPTTG mice

**ID**# **and Tissue**	**PCNA**	**CD31**
WT Ovary	1%	+
TgPTTG #434 Ovary	35%	+ +
TgPTTG #436 Ovary	5%	+ +
WT Cervix	1%	NA
TgPTTG #286 Cervix	5%	NA
TgPTTG #436 Cervix	5-10%	NA
WT Fallopian Tube	0%	NA
TgPTTG #365 Fallopian Tube	25%	NA

PCNA is percent of positively stained nuclei vs. total. + = < 25%, + + = 25-75%, + + + = > 75%.

**Figure 8:**
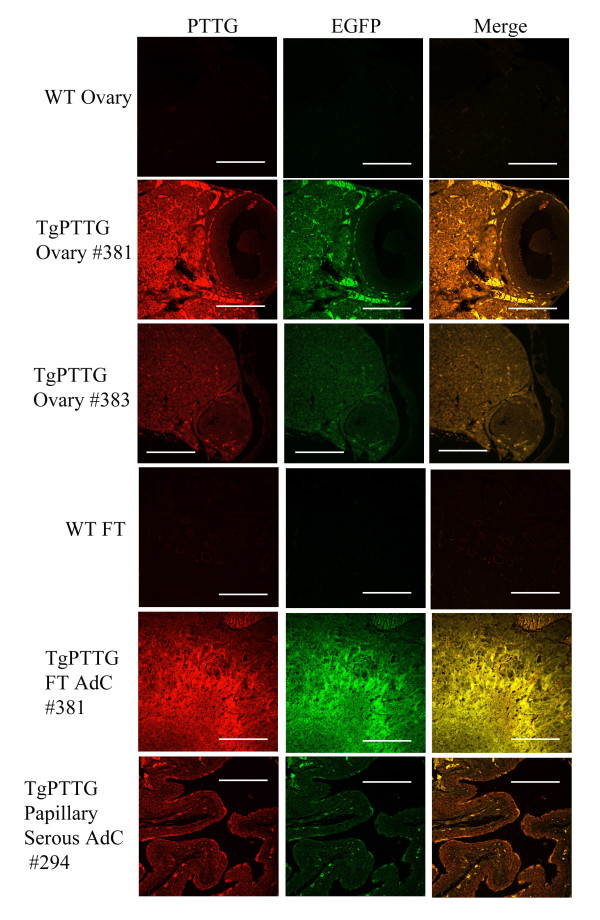
**Immunofluorescence of TgPTTG 10 months old mice for PTTG**-**EGFP protein expression.** PTTG antibody was used for immunostaining of PTTG protein and detected with Alexa 594 fluorescent antibody (red). Images were acquired using confocal microscopy. WT = wild type, FT = fallopian tube, AdC = adenocarcinoma. White bar indicates 50 μm.

**Figure 9:**
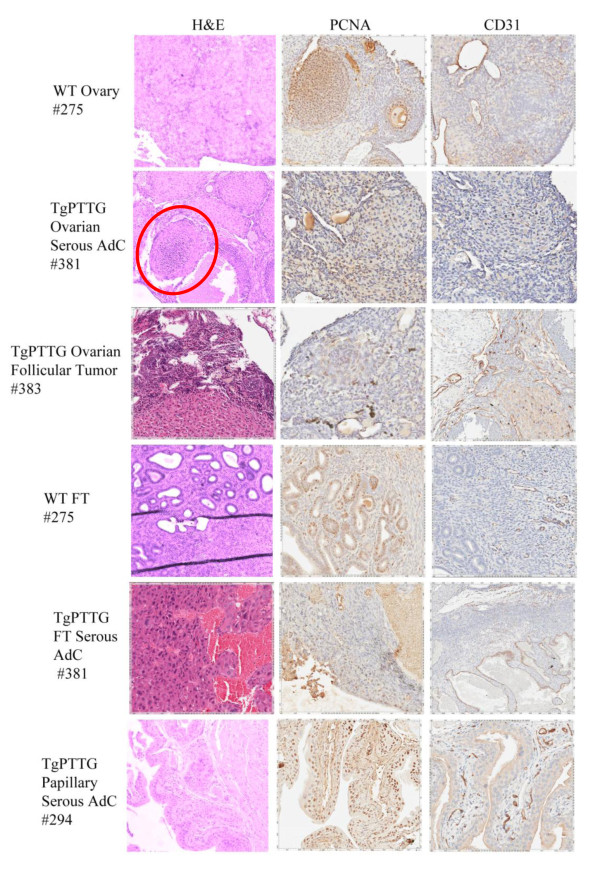
**Immunohistochemistry of 10 months TgPTTG mice with cancer compared to age**-**matched WT mice.** PCNA and CD31 antibodies were incubated overnight then visualized with DAB (brown color). Red circle indicates *in situ* tumor.

Ten months old TgPTTG group included 10 females and was compared to 13 WT age-matched controls. PTTG-EGFP expression was confirmed by immunofluorescence (Figure 8). One female developed a pre-cancerous serous cyst adenoma of the ovary. Another developed serous adenocarcinoma of the ovary and fallopian tube (#381, Figure 9), while an additional female developed a tumor of ovarian follicular cells (#383, Figure 9). PCNA staining was 5-10% in both TgPTTG ovaries and 15% in age-matched WT controls which was localized to the follicles (Figure 9). CD31 staining was similar between WT ovaries and TgPTTG ovaries (1+, Figure 9). Interestingly, PTTG expression was higher in the female that developed serous adenocarcinoma vs. the female with the follicular cells tumor (Figure 8). Additionally, 1 out of 20 (5%) of TgPTTG mice developed an ectopic tumor diagnosed as a papillary serous adenocarcinoma that was 75% positive for PCNA and had significant CD31 staining (3+, Figure 9). Two other females (20%) showed atypia of epithelial cells of the fallopian tube. A summary of PCNA and CD31 staining patterns is shown in Table 3. All other tissues were devoid of visible tumors.

**Table 3:** Summary of PCNA and CD31 staining in 10 months old WT and TgPTTG mice

**ID**# **and Tissue**	**PCNA**	**CD31**
WT Ovary	15%	+ +
TgPTTG #381 Ovary	5-10%	+
TgPTTG #383 Ovary	5-10%	+
WT Fallopian Tube	0%	+
TgPTTG #381 Fallopian Tube	10%	+ + +
TgPTTG #294 PS AdC	75%	+ + +

PCNA is percent of positively stained nuclei vs. total. + = < 25%, + + = 25-75%, + + + = > 75%. PS AdC = papillary serous adenocarcinoma.

### Transgene copy number analysis

Although unlikely, the transgene copy number could have been unstable, and therefore vary among offspring. To exclude this possibility, transgene copy number was assessed to analyze differences between TgPTTG that developed cancer and those that did not. A standard curve was generated using the N_3_ vector containing PTTG (Additional file 1: Figure S 1A). By calculating the size of the vector, a copy number could be assessed to the standard curve. Then, by taking 100 ng of genomic DNA isolated from the tail clip, the gene copy number was extrapolated from the standard curve as 100 ng of DNA has been reported to yield 1.67 x 10^4^ diploid cells [29]. We found no difference in transgene copy number between TgPTTG mice that developed cancer and those that did not develop cancer (Additional file 1: Figure S 1B).

### Crossbreeding of TgPTTG with p53^+/−^ mice and histology

P53 mutant mice were produced by a targeted neo cassette insertion into the p53 locus in the laboratory of Dr. Tyler Jacks at the Center for Cancer Research, Massachusetts Institute of Technology [30] and are commercially available through the Jackson Laboratory. P53^+/−^ mice have been reported to have an 18% incidence of adenocarcinomas and a 56% incidence of sarcomas over a period of 17 months [31]. Based on the 56 mice we observed (45 p53^+/−^ and 11 p53^−/−^), four p53^+/−^ mice (8.9%) developed sarcomas between 3 to 11 months, while nine p53^−/−^ (81.8%) developed lymphoma and sarcomas at age 9 to 12 weeks before succumbing to their disease.

**Figure 10:**
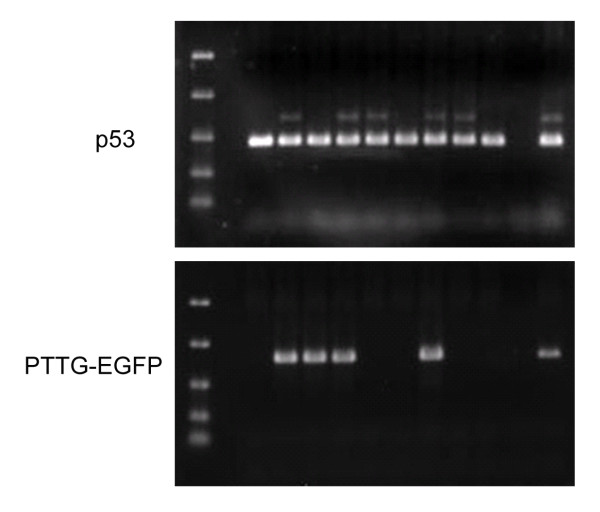
**Representative genotyping of offspring from TgPTTG crossbred to p53**^+/−^**mice.** Lane 1, ladder; lane 2, empty; lane 3–9, individual mice samples (ID# 487–495); lane 10 is negative control of PCR reaction mix without template DNA; and lane 11, positive control. p53^+/−^ mice were genotyped using recommended primers from Jackson labs resulting in a robust 400 kb band for p53^+^ and a weaker 600 kb band for p53^-^. Mice were then cross-referenced to PTTG-EGFP genotype.

**Figure 11:**
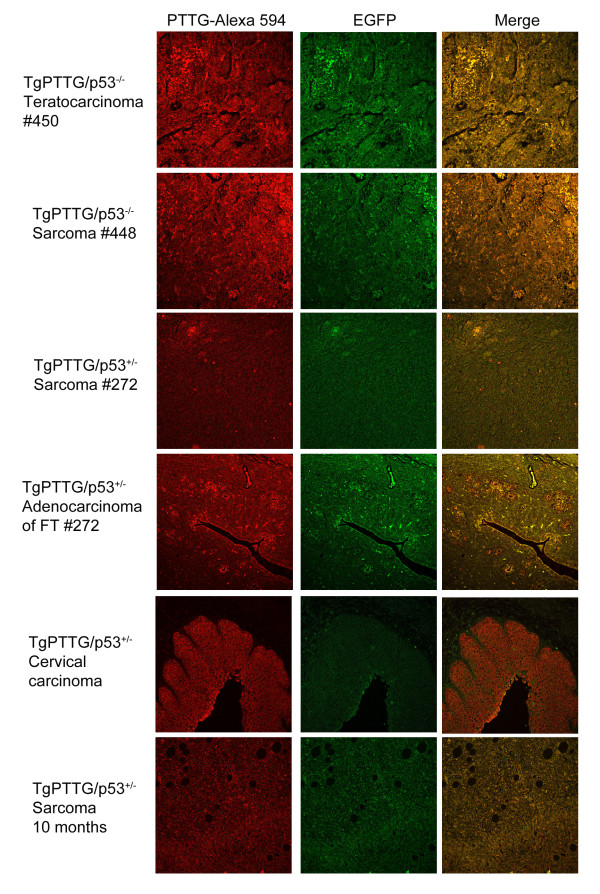
**Immunofluorescence of TgPTTG**/**p53**^+/−^**mice for PTTG**-**EGFP protein expression.** PTTG antibody was used for immunostaining of PTTG protein and detected with Alexa 594 fluorescent antibody (red). Images were acquired using confocal microscopy. FT = fallopian tube.

**Figure 12:**
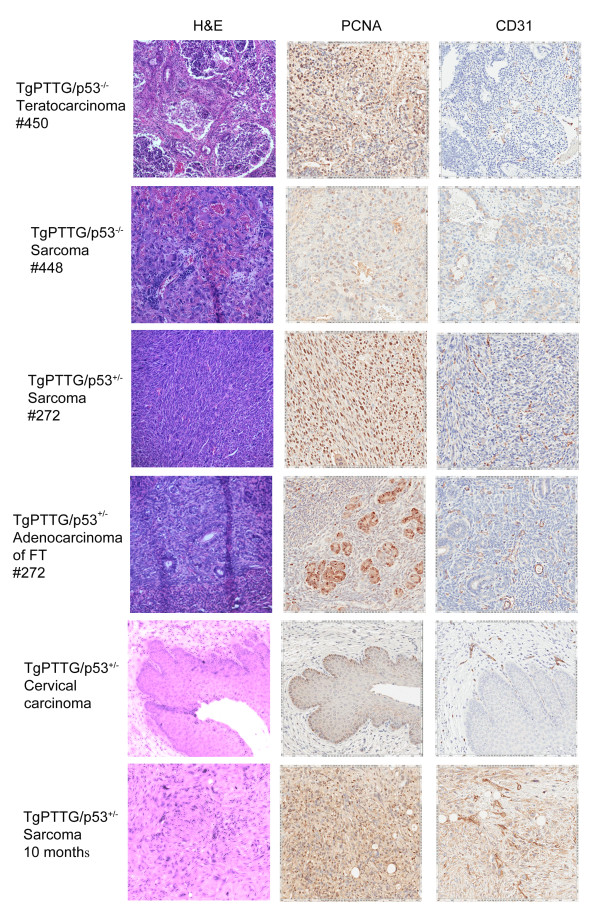
**Immunohistochemistry of TgPTTG**/**p53**^+/−^**mice at various ages. ** PCNA and CD31 antibodies were incubated overnight then visualized with DAB (brown color).

Due to the short lifespan of p53^−/−^ mice, p53^+/−^ mice were chosen for crossbreeding to investigate if a partner gene would increase the incidence, decrease the onset time, and/or possibly change the type of cancer in TgPTTG mice. Two male TgPTTG mice from line #71309 were crossbred with p53^+/−^ females to maximize offspring, resulting in generation of F_2_ (Figure 10). Accuracy of p53 status of TgPTTG/p53^−/−^ and TgPTTG/p53^+/−^ was confirmed by sequencing genomic DNA. PTTG-EGFP expression was analyzed by immunofluorescence and was higher in TgPTTG/p53^−/−^ tumors than TgPTTG/p53^+/−^ (Figure 11). Due to tumor burden, three mice necessitated premature euthanization. Of the three, one TgPTTG/p53^−/−^ mouse (#450) developed two teratocarcinomas at 7 weeks of age, while a second mouse developed lymphoma and two sarcomas at 11 weeks of age (#448, Figure 12). Comparatively, p53^−/−^ mice develop lymphoma and sarcomas at 12 weeks (9 of 11). The third mouse was TgPTTG/p53^+/− ^between TGPTTG and p53+ that developed adenocarcinoma of the fallopian tube and leiomyosarcoma with mitotic figures at 7 months of age (#272, Figure 12). PCNA and CD31 analysis was performed in these tissues. PCNA was 90% in the teratocarcinoma as well as TgPTTG/p53^−/−^ sarcoma and 99% in the TgPTTG/p53^+/−^ leiomyosarcoma (Figure 12). All tumors showed CD31 staining of 1+ (Figure 12).

TgPTTG/p53^+/−^ mice aged to 8 months included 10 females. Of the TgPTTG/p53^+/−^ females aged to 8 months, 4 out of 10 (40%) had severe dysplasia of the cervix resulting in carcinomas *in situ* (Figure 12) along with 1 out of 10 (10%) developing a sarcoma. PCNA staining for cervical carcinomas *in situ* showed 10% positive area with reverse maturation and 2+ microvessel formation. PCNA showed 99% positive area in all sarcomas, congruent with the highly aggressive nature of this tumor type.

TgPTTG/p53^+/−^ mice aged to 10 months included 8 females. Five out of 8 (63%) females showed focal to severe cervical dysplasia resulting in carcinomas *in situ*, and 3 out of 8 (38%) showed dilation of the fallopian tube. Two out of 8 (25%) developed high grade leiomyosarcomas (Figure 12). All TgPTTG/p53^+/−^ sarcomas were 99% positive for PCNA and had significant microvessel formation (CD31 staining 3+) determined by CD31 (Figure 12). Comparatively, p53^+/−^ mice developed sarcomas between 11 to 12 months of age (2 of 28, 7%).

**Figure 13:**
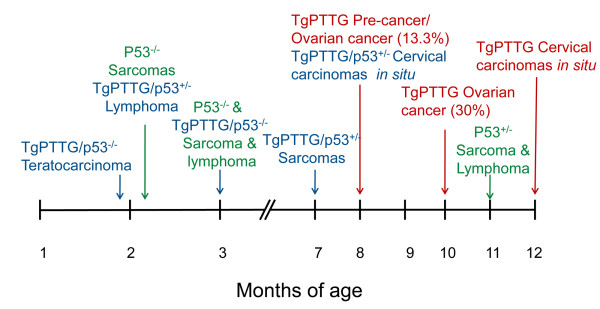
**Timeline summary of pre**-**cancerous and cancer results for TgPTTG** (**red**), **p53**^−/−^ (**green**), **p53**^+/−^ (**green**), **and TgPTTG**/**p53**^+/−^ (**blue**).

The timeline of tumor developments in TgPTTG, p53^+/−^, and TgPTTG/p53^+/−^ is summarized in Figure 13.

## Discussion

PTTG has been shown in pituitary tumors to have the properties of a transforming gene activated during the early stages of neoplastic transformation, changing the cells from a normal phenotype to hyperplastic [13]. Based on this information, a transgenic mouse expressing PTTG under the control of αGSU promoter to target the expression to the gonadotroph cells of the pituitary was created [23]. This resulted in gonadroph hyperplasia and microadenomas with plurihormal hyperplasia, accompanied by prostatic and seminal vesicle hyperplasia, demonstrating that PTTG was a functional transforming oncogene. Since PTTG is a prominent oncogene in pituitary tumors, many investigators have identified PTTG in several other endocrine-related tumors. Its expression has been detected in endometriod carcinomas and in hyperplastic endometria [32]. In addition to being cloned from ovarian tumors, PTTG has been identified in various ovarian tumor tissues but not in normal ovary [11]. In addition, a mouse xenograft model of ovarian cancer showed that PTTG was crucial for tumor development [19]. Therefore, previously our lab developed a transgenic mouse model that expresses human *PTTG* cDNA under the control of MISIIR. These mice presented with an increased mass of the corpus luteum as well as an increase in the serum LH and testosterone. However, despite PTTG expression in the ovary and testes, these animals developed cystic glandular hyperplasia but failed to develop visible ovarian adenocarcinomas [22], possible due to a weak promoter that was unable to produce the required level of PTTG protein to initiate tumorigenesis. However, we also asked if a partner gene was necessary to complete tumorigenesis as it often requires multiple gene mutations including activation or amplification of oncogenes, increased growth factors and their receptors, and/or inactivation of tumor suppressor genes, and therefore PTTG expression by itself may not be sufficient to drive tumorigenesis and may require a partner gene [24].

Therefore, in our current study to understand the tumorigenic potential of PTTG, we generated a transgenic mouse that constitutively overexpresses PTTG under the control of the non-specific CMV promoter to strengthen PTTG expression as the level of PTTG is critical for tumorigenesis [19,33]. By using this approach, we were able to generate ovarian tumors in transgenic animals as early as 8 months of age (Figure 7) and continued to observe them at 10 months (Figure 9), albeit at a low incidence of ~17%. It is interesting that although the CMV promoter is a non-specific promoter, ovarian and fallopian tube tumors and papillary serous adenocarcinomas were the only observed tumor types, perhaps due to the site of integration. However, PTTG also has transactivation activity through the presence of Src-homology 3 (SH3) domains located in the proline-rich regions of the C-terminus [34]. As such, PTTG increases the excretion of bFGF [11], which itself can increase ovarian cancer invasion [35]; therefore, PTTG itself may not directly induce tumorigenesis but acts indirectly on tissues expressing a higher level of bFGF, such as the ovary, fallopian tube, and cervix [36-38] to increase angiogenesis and stimulate expression of other activator genes, such as Ets-1 and urokinase-type plasminogen activator to promote tumor progression.

 Since our incidence of tumor formation was ~17%, we cross-bred our TgPTTG mice with p53 mutant (p53^+/−^) mice with the assumption that p53^+/−^ would enhance the incidence. Using this approach, TgPTTG/p53^+/−^ animals resulted in earlier tumor development than PTTG or p53^+/−^ alone (Figure 13) and increased the incidence of high grade leiomyosarcomas from 7% in p53^+/−^ mice to 14% in TgPTTG/p53^+/−^. We also noted a significant incidence of cervical carcinomas of 63% in TgPTTG/p53^+/−^. Most cases of cervical cancer in women are due to human papilloma virus (HPV) infections that cause inactivation of p53 and Rb, mutations that mainly affect the squamous epithelium along the transformation zone as HPV infections require cells that are proliferating [39]. In our TgPTTG mice, we noticed a reverse maturation of the basal squamous epithelium coupled to dysplasia. As PTTG increases cell proliferation as observed by PCNA staining, PTTG could serve to prime the basal epithelium, which could make these particular cells more susceptible to further neoplastic transformation due to alterations of additional genes, such as p53. We also noted teratocarcinomas in TgPTTG/p53^−/−^. While little is known about the genetic contributions to teratocarcinomas, the field theory states that normal germ cells that are placed in an environment that allows expression of the cancer phenotype have the potential to become cancerous [40]. Constitutive overexpression of PTTG may alter the micro environment through enhanced secretion of growth factors, and thus allow normal cells to proliferate more rapidly causing transformation of normal cells to become tumorigenic leading to the formation of tumors, such as teratocarcinomas.

## Conclusions

In our studies, we showed that PTTG is a functional oncogene that is capable of initiating transformation of normal tissue to dysplastic. Furthermore, a high expression level of PTTG was capable of inducing tumorigenesis in the ovary and other tissues leading to tumorigenesis. Coupling of TgPTTG with mutant p53 led us to conclude that the early neoplastic events (normal to dysplastic) were independent of p53 as the additional mutation did not enhance incidence of ovarian cancer; however overexpression of PTTG and loss of p53 function reduced the time of development of certain cancers, including sarcoma.

## Abbreviations

αGSU: Alpha-subunit of glycoprotein hormone; bFGF: Basic fibroblast growth factor; IL8: Interleukin 8; FVB: Friend Virus B-Type; MISIIR: Müllerian inhibitory substance type II receptor; PTTG: Pituitary tumor transforming gene; Rb^+/−^: Heterozygous deletion of retinoblastoma; SH3: Src-homology 3; TgPTTG: PTTG transgenic mice; VEGF: Vascular endothelial growth factor.

## Competing interests

The authors declare no competing interests.

## Authors' contributions

MYF designed and performed experiments, analyzed and interpreted the data, and drafted the manuscript. HF analyzed and interpreted the data. SSK conceptualized and designed experiments, analyzed and interpreted the data, and helped in drafting the manuscript. All authors read and approved the final manuscript.

## Pre-publication history

The pre-publication history for this paper can be accessed here:

http://www.biomedcentral.com/1471-2407/12/532/prepub

## Supplementary Material

Additional file 1 **Figure S1.** Transgene copy number analysis of TgPTTG mice that developed cancer and TgPTTG mice that did not. **(A)** Standard curve generated from N_3_ cloning vector containing PTTG in the multiple cloning site. **(B)** Real-time PCR analysis of transgene copy number extrapolated from standard curve plotted as average ± SD. TgPTTG mice were selected from 8 months – 10 months. N = 3 for no cancer, N = 6 for cancer. Click here for file
