# Supplementary figures and images for: Tumorigenic potential of pituitary tumor transforming gene (PTTG) in vivo investigated using a transgenic mouse model, and effects of cross breeding with p53 (+/−) transgenic mice
Source: BMC Cancer. 2012 Nov 20;12:532. doi: 10.1186/1471-2407-12-532 (PMC3530432; doi:10.1186/1471-2407-12-532)

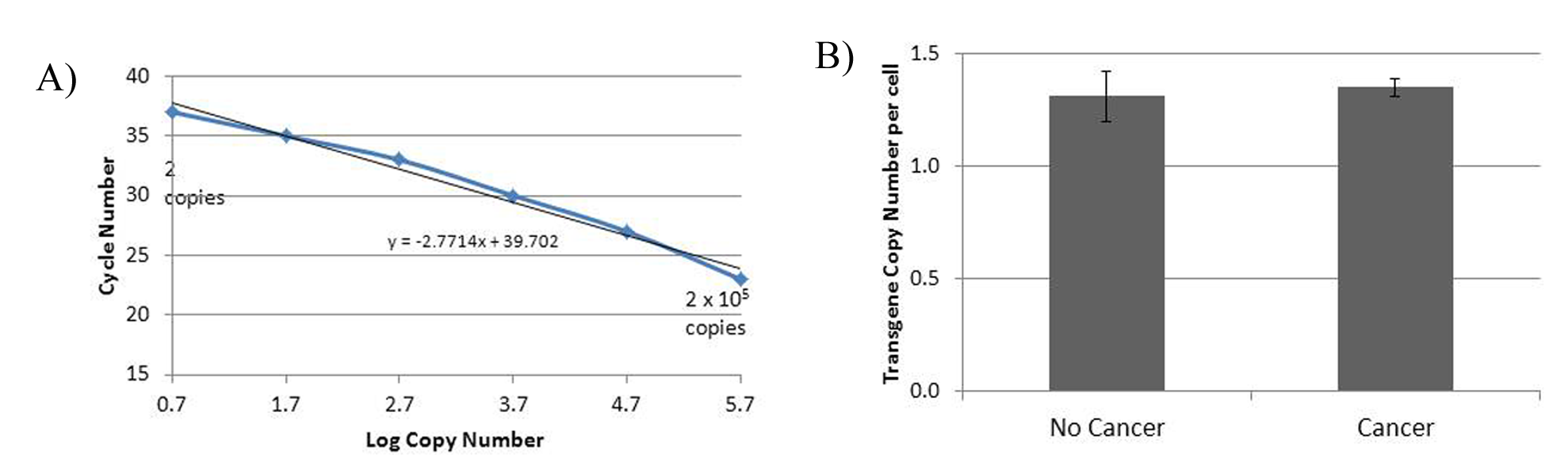

Supplement: Additional file 1 — Figure S1. Transgene copy number analysis of TgPTTG mice that developed cancer and TgPTTG mice that did not. (A) Standard curve generated from N3 cloning vector containing PTTG in the multiple cloning site. (B) Real-time PCR analysis of transgene copy number extrapolated from standard curve plotted as average ± SD. TgPTTG mice were selected from 8 months – 10 months. N = 3 for no cancer, N = 6 for cancer. [file 1471-2407-12-532-S1.tiff]
